# Current patterns of next-generation sequencing testing in biliary tract cancer patients in Spain

**DOI:** 10.1007/s12094-026-04397-3

**Published:** 2026-05-28

**Authors:** Jorge Adeva, Alfredo Carrato, Teresa Macarulla

**Affiliations:** 1 Department of Medical Oncology, Hospital Universitario, 12 de Octubre, Madrid, Spain; 2 Department of Medicine, IRYCIS (Ramón y Cajal Institute for Health Research), Madrid, Alcalá University, Madrid, Spain; 3 Department of Medical Oncology, Clínic Barcelona Comprehensive Cancer Center, Hospital Clínic de Barcelona, Barcelona, Spain; 4 Translational Oncology in Upper Gastrointestinal Cancer Group, IDIBAPS, Barcelona, Spain; 5 Department of Medicine, University of Barcelona, Barcelona, Spain

**Keywords:** Biliary tract cancer, Cholangiocarcinoma, Molecular diagnostics, Next-generation sequencing (NGS), Precision medicine, Targeted therapies

## Abstract

**Purpose:**

Biliary tract cancer (BTC), which includes cholangiocarcinoma (CCA) and gallbladder cancer (GBC), presents high levels of molecular heterogeneity and a poor prognosis. Next-generation sequencing (NGS) is crucial for identifying genomic alterations and adequate targeted therapies, but its integration into clinical practice varies by region. This study assesses how NGS is implemented in Spain, focusing on its use, barriers, and clinical impact on managing BTC.

**Methods:**

This study was based on a national survey of healthcare professionals involved in the diagnosis and treatment of BTC.

**Results:**

Seventy-six experts participated in the general analysis, and 58 participated in the BTC-specific analysis. Among the respondents, 95% reported access to NGS in oncology practice, and 93% reported its use in BTC. FGFR2 and IDH1/2 were the most frequently requested biomarkers (both 91%), whereas TMB (22%) and expanded panels were less commonly used. Thirty-two of 50 respondents requested NGS in advanced disease (64%), as per the responses to this specific question, which is consistent with current therapeutic indications. Thirty-four of 48 respondents performing NGS (71%) reported access to specialized genetic counseling. The perceived therapeutic impact varied: many estimated that NGS influenced treatment decisions in 20–40% of cases, whereas a subset reported that it influenced treatment decisions in more than 60% of cases.

**Conclusions:**

The adoption of NGS in BTC management in Spain has increased alongside the availability of targeted therapies. The respondents reported broad access to NGS and frequent use of key biomarkers, mainly in advanced disease. Variability in biomarker selection, timing, and access to counseling reflects differences in local practice. Initiatives that support harmonized diagnostic pathways may further strengthen precision oncology in BTC.

**Supplementary Information:**

The online version contains supplementary material available at https://doi.org/10.1007/s12094-026-04397-3.

## Introduction

Biliary tract cancer (BTC) primarily includes cholangiocarcinoma (CCA) and gallbladder cancer (GBC). CCA originates in the biliary epithelium and is anatomically classified into intrahepatic, perihilar, and distal subtypes, the latter two which are considered extrahepatic [1–3]. Despite its relatively low incidence, CCA is highly aggressive and is associated with poor prognosis and an increasing healthcare burden [4].

Globally, the incidence ranges between 0.3 and 2 cases per 100,000 inhabitants, with geographic variability reflecting environmental and genetic factors [5]. In Spain, BTC represents approximately 3% of digestive malignancies and remains associated with substantial morbidity and mortality [6].

Mortality rates from CCA have been rising steadily worldwide, and Spain is among the countries with the highest rates in Europe, ranging between 1.7 and 2.3 per 100,000 inhabitants [5, 7].

Diagnosis remains challenging because of the nonspecific clinical presentation, resulting in advanced stage detection in a large proportion of patients [8]. Diagnosis relies on imaging techniques, especially computed tomography (CT) scans with or without magnetic resonance imaging (MRI), and histological analysis when surgery is not possible and when tumor markers such as CA 19–9 are present [9, 10]. Complete surgical resection is the only cure; however, most patients relapse after surgery or present with advanced disease, requiring palliative systemic therapy [11].

In recent years, next-generation sequencing (NGS) has emerged as an essential tool for the molecular characterization of BTC, enabling the identification of actionable genomic alterations and facilitating precision oncology strategies [12]. Molecular profiling studies have demonstrated that approximately 30–50% of patients with CCA harbor potentially targetable alterations, including FGFR2 fusions, IDH1 mutations, HER2 alterations, and BRAF mutations [13].

The implementation of NGS has significantly improved the detection of actionable alterations and therapeutic stratification across tumor types, supporting its incorporation into routine oncologic practice [14].

International guidelines, including recommendations from the European Society for Medical Oncology (ESMO), advocate routine molecular profiling using NGS in patients with advanced BTC to guide treatment selection and clinical trial enrollment [13, 15]. However, real-world implementation remains heterogeneous, with barriers including infrastructure limitations, resource availability, and variability in access to specialized molecular diagnostics [16].

Understanding real-world implementation patterns is essential for identifying gaps and optimizing precision oncology strategies. Therefore, this study evaluates the availability, utilization patterns, and clinical integration of NGS techniques in the management of CCA in Spain on the basis of a national survey of healthcare professionals involved in BTC care.

## Materials and methods

### Study design

A cross-sectional, multicenter observational study was conducted using a national survey of healthcare professionals involved in the clinical management of digestive cancers.

The present analysis focuses specifically on the BTC module, including CCA and GBC. The findings were reported in accordance with the Strengthening the Reporting of Observational Studies in Epidemiology (STROBE) checklist [17].

### Setting

Data were collected from March–May, 2025, using a structured questionnaire that was distributed electronically. Participation in the survey was open, voluntary, and unpaid. Only responses from centers located in Spain were included in the final analysis.

### Participants

The participants included medical oncologists, pathologists, molecular biologists, hepatologists, and other specialists involved in the diagnosis and treatment of BTC. Eligibility of the participants was defined by their active involvement in the clinical management or molecular diagnosis of digestive malignancies.

### Data collection

The questionnaire collected information regarding the availability of NGS at participating centers, the biomarkers included in the molecular testing panels, the timing of NGS testing, access to genetic counseling, barriers to implementation, and involvement in research and clinical trials. Both single-choice and multiple-choice questions were included.

### Potential sources of bias

Potential sources of bias included selection bias, as centers with greater access to molecular testing may have been more likely to participate, and reporting bias related to self-reported clinical practices. To reduce these biases, we included experts from various autonomous communities and different levels of care. The distribution of respondents across autonomous communities was uneven, with some regions having only one or two participants. As a result, regional estimates, especially those at 100%, may be affected by small sample sizes and should be interpreted with caution.

### Data sources and measurement methods

Information was gathered using a questionnaire specifically developed for this study. The questionnaire included predefined domains covering NGS availability, biomarker assessment, clinical integration, and access to molecular diagnostics.

### Sample size

No formal sample size calculation was conducted because of the exploratory nature of the study. A convenience sampling approach was used.

### Statistical analysis

Descriptive statistics were conducted for all the variables, and absolute frequencies and percentages were calculated. Statistical analyses were performed using Stata^®^ 18.0 software (StataCorp, College Station, TX, USA).

### Ethical considerations

This study did not require Ethics Committee review because it did not collect patient identification data or sensitive information. Participants provided electronic informed consent prior to participation.

## Results

All the results were derived from the survey respondents and represent only the practices of the participating centers. They should not be extrapolated to all institutions within each Spanish autonomous community.

### Overall results of NGS use in oncology in Spain

Among the 76 experts who participated in the general analyses, 72 (95%) reported that NGS was implemented at their respective centers, either by using their own platforms or by referring patients to external laboratories. Among the respondents from each autonomous community, 100% reported access to NGS in Andalusia, Aragon, Asturias, Cantabria, the Valencian Community, Galicia, the Balearic Islands, the Canary Islands, La Rioja, Navarre, the Basque Country, and the Region of Murcia. In contrast, respondents from Castile-La Mancha (67%), Catalonia (93%), and the Community of Madrid (93%) showed partial adoption of NGS. Overall, the majority of participants who reported their specialty indicated that they belonged to medical oncology departments. In the Community of Madrid, three professionals from Hospital Clínico San Carlos completed the survey: two (67%) from medical oncology and one (33%) from pathology. In Galicia, among the respondents from the five participating centers, four (80%) reported medical oncology as their specialty, and one (20%) reported pathology.

### Specific use of NGS in CCA and GBC

Among the 58 experts in the BTC survey, 54 (93%) reported that their centers conducted NGS studies for CCA and GBC.

The geographical distribution of access reveals notable differences among respondents. In several autonomous communities, all the respondents reported having access to NGS, resulting in values of 100% for regions such as Andalusia, Aragon, Asturias, Cantabria, Castile-La Mancha, Castile and León, the Valencian Community, the Balearic Islands, the Canary Islands, La Rioja, Navarre, the Basque Country, and Murcia. In regions where a greater number of respondents participated, such as Catalonia (90%), the Community of Madrid (82%), and Galicia (75%), some respondents indicated a lack of access, resulting in comparatively lower proportions. The geographical distribution of the participating centers is shown in Supplementary Fig. 1.

Among the respondents included in the survey, the communities with the greatest number of responding centers were the Community of Madrid (11 centers) and Catalonia (10 centers), followed by the Valencian Community and Andalusia (6 centers each) and Castile and León (5 centers). Galicia contributed four participating centers, and the Basque Country contributed three. Regions with two participating centers included Cantabria, Navarre, and the Canary Islands, while one participating center was reported from Aragon, Asturias, Castile-La Mancha, the Balearic Islands, La Rioja, and the Region of Murcia.

### Biomarkers requested and NGS techniques used in CCA and GBC

**Fig. 1:**
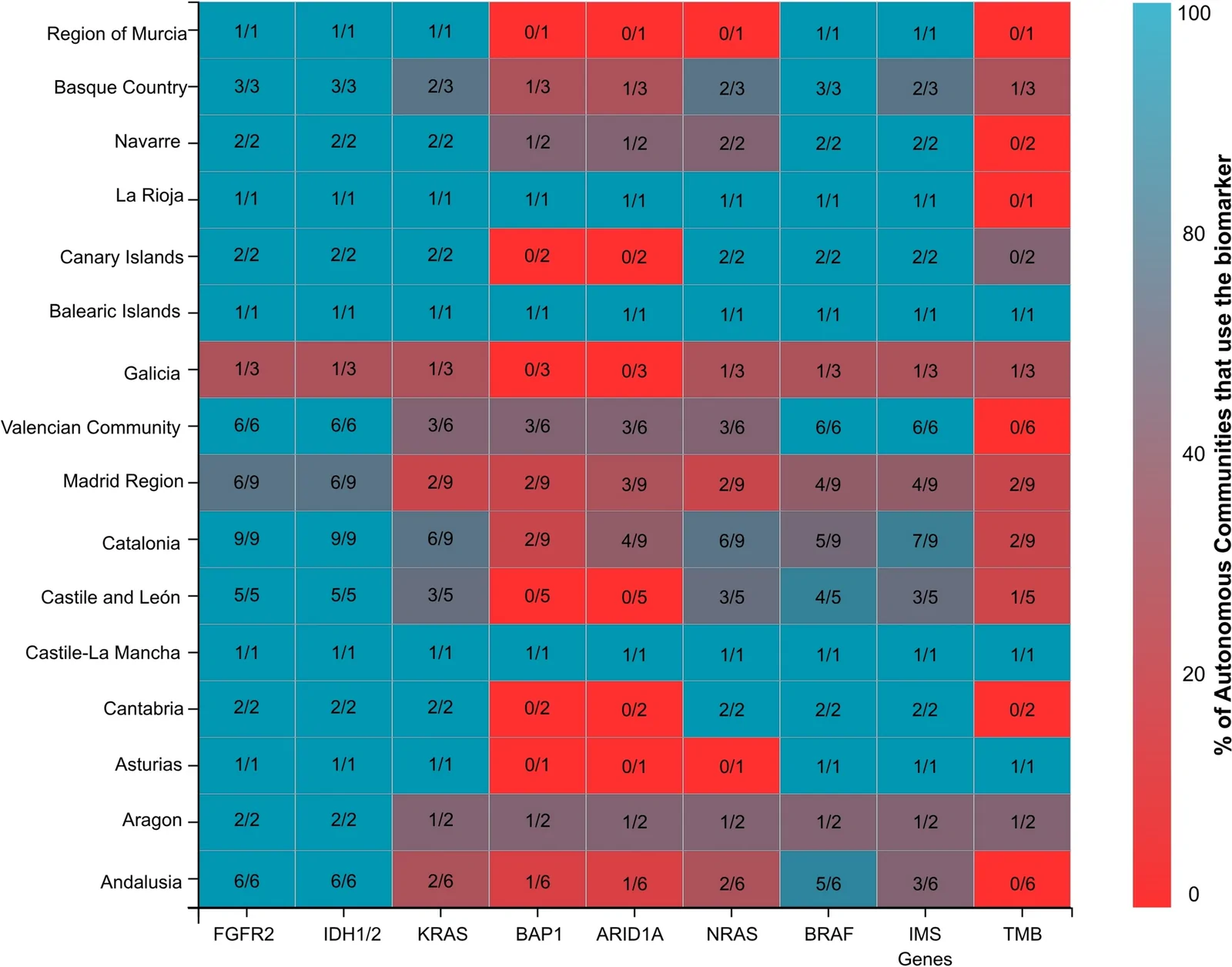
Territorial variability in the use of biomarkers and of next-generation sequencing (NGS) techniques in cholangiocarcinoma and gallbladder cancer in Spain. All the percentages are calculated on the basis of the number of respondents from each community and therefore reflect only the practices of the participating centers, not all the institutions in that region. Values represent the percentage of respondents in each autonomous community who reported using each biomarker. Each cell also shows the absolute number as n/N, where N is the number of respondents for that biomarker in the community. Because respondent numbers vary by biomarker and region, results are unweighted and should be interpreted with caution, especially in communities with few participants

NGS-based biomarker determination in the CCA/GBC showed notable variation among the 54 respondents who provided information on biomarker use (Fig. 1).

FGFR2 fusions and IDH1 mutations were the most consistently requested biomarkers, each reported by 49 of 54 respondents (91%), confirming their widespread incorporation into routine molecular testing. KRAS and BRAF mutations were also frequently included in NGS panels and were reported by 31 (57%) and 40 (74%) respondents, respectively.

Biomarkers commonly associated with extended or comprehensive panels, such as mutations in BAP1 (14/54, 26%), ARID1A (17/54, 32%), and NRAS (28/54, 52%), were reported less frequently overall.

Mismatch repair (MMR) genes (MLH1, MSH2, MSH6, PMS2, EPCAM) were widely reported, with 38 of 54 respondents (70%) indicating their use. In contrast, tumor mutational burden (TMB) was requested by 12 respondents (22%), making it one of the least commonly used biomarkers.

HER2 alterations assessed by NGS were also frequently reported, with 43 respondents (80%) indicating their inclusion in molecular testing panels.

The use of expanded molecular panels was limited, with only isolated respondents reporting their use. Notably, this practice was reported exclusively in the Community of Madrid, where a minority of respondents (2/9; 22%) indicated using expanded panels.

### Clinical timing of NGS requests

**Fig. 2:**
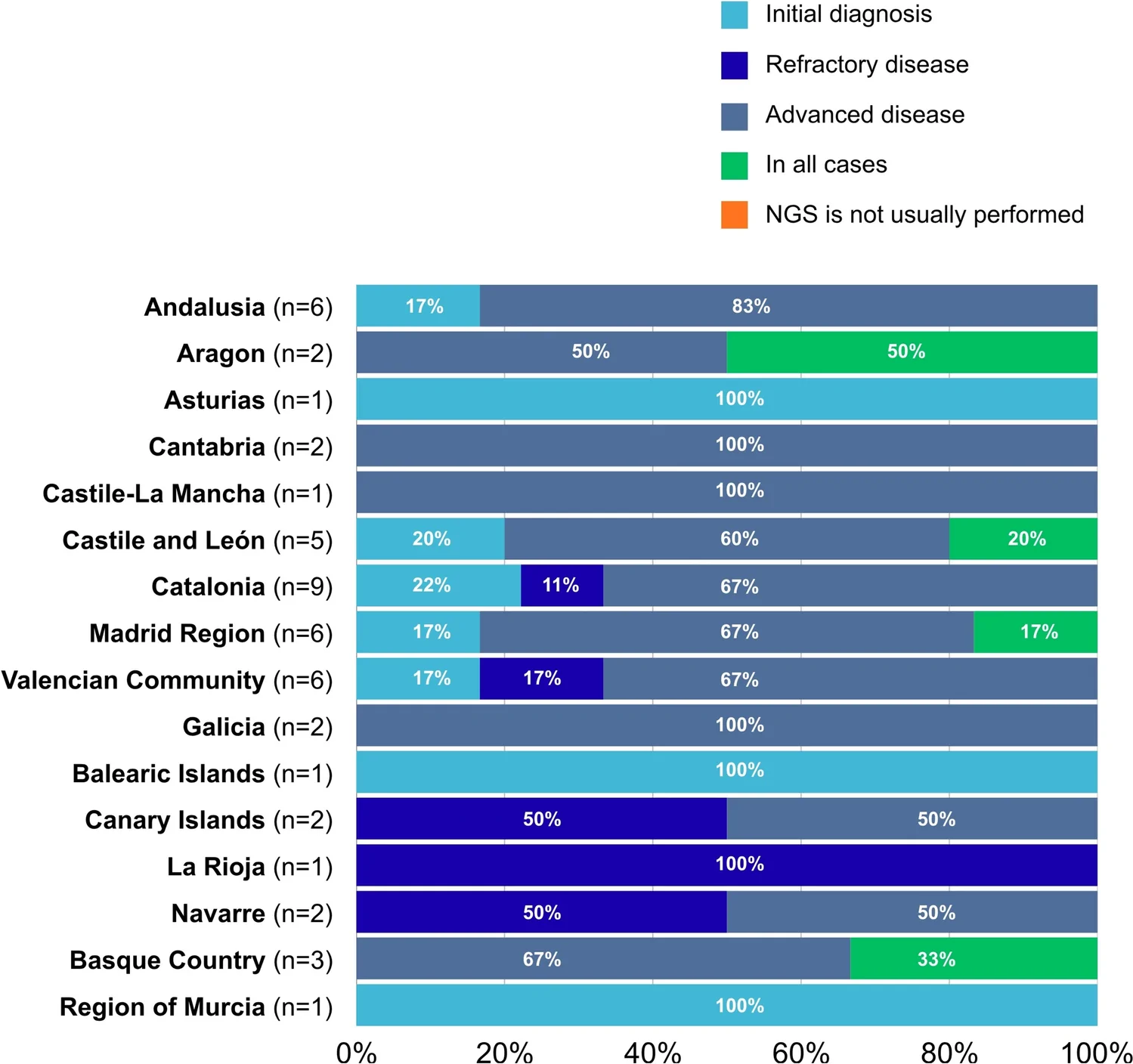
Management stage of cholangiocarcinoma and gallbladder cancer for which next-generation sequencing (NGS) analysis is requested according to the autonomous community. All the percentages are calculated on the basis of the number of respondents from each community and therefore reflect only the practices of the participating centers, not all the institutions in that region. All percentages are calculated based on the number of respondents from each autonomous community, as indicated by n in the figure. The data reflect only the practices of the participating centers, not all institutions in each region

Among the 50 respondents who provided information on the clinical timing of NGS, 32 (64%) indicated that NGS is requested at advanced stages of disease (Fig. 2). However, the survey did not further specify whether NGS testing in advanced disease was performed before, during, or after first-line systemic treatment.

The use of NGS at initial diagnosis in resectable disease was less common, as reported by nine respondents (18%) from different autonomous communities, including Andalusia, Asturias, Castile and León, Catalonia, the Community of Madrid, the Valencian Community, the Balearic Islands, and Murcia.

Requests for NGS in the context of refractory disease after progression to standard therapies were infrequent and reported by only five respondents (10%) across several communities, including Catalonia, the Valencian Community, the Canary Islands, La Rioja, and Navarre.

A minority of respondents (4/50, 8%) indicated that NGS was performed in all patients with CCA regardless of clinical stage. These responses came from Aragon, Castile and León, the Community of Madrid, and the Basque Country.

### Clinical indications for NGS, access barriers, and usefulness in therapeutic decision-making

**Fig. 3:**
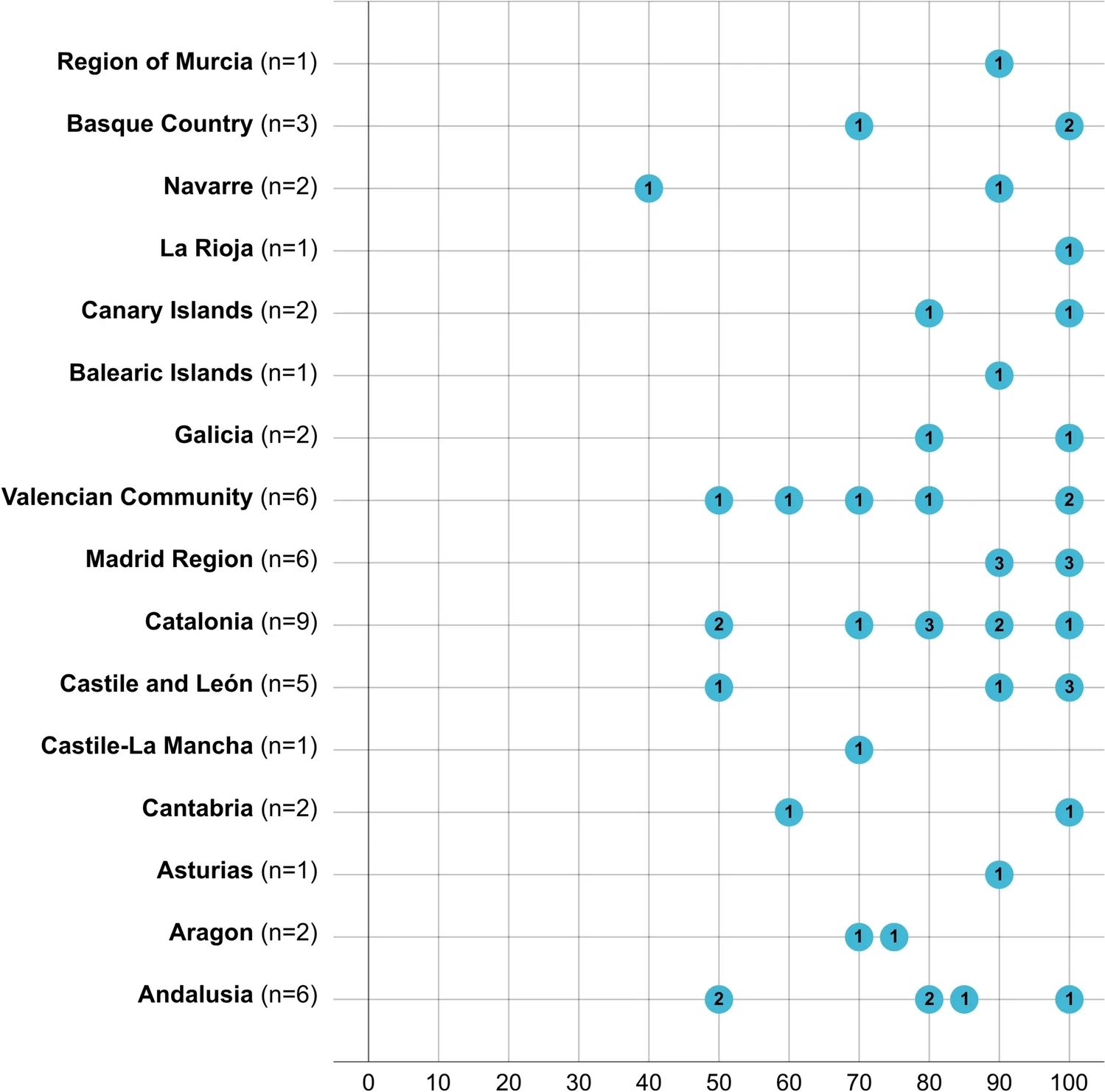
Estimated percentage of patients with cholangiocarcinoma and gallbladder cancer considered clinically eligible for next-generation sequencing (NGS). The number within each point indicates the number of respondents with that same answer. The total number of respondents per autonomous community is shown in parentheses

On the basis of the responses from the 50 participants, the proportion of patients with CCA and GBC who were considered as candidates for NGS was generally high, with most respondents indicating that between 70 and 100% of their patients met the clinical criteria for testing (Fig. 3). Variability was noted both between and within certain autonomous communities. The highest response rates, ranging from 90 to 100%, were consistently found in the Community of Madrid, Castile and León, La Rioja, the Balearic Islands, the Region of Murcia, and Asturias, as well as among some respondents in Catalonia, Galicia, Navarre, the Basque Country, the Canary Islands, Andalusia, and Valencian Community. Some communities showed greater response dispersion. For instance, Navarre reported estimates of 40% and 90%, while Catalonia, Andalusia, and Valencian Community ranged from 50 to 100%. Intermediate values were observed in Cantabria, Aragon, and Castilla-La Mancha.

**Fig. 4:**
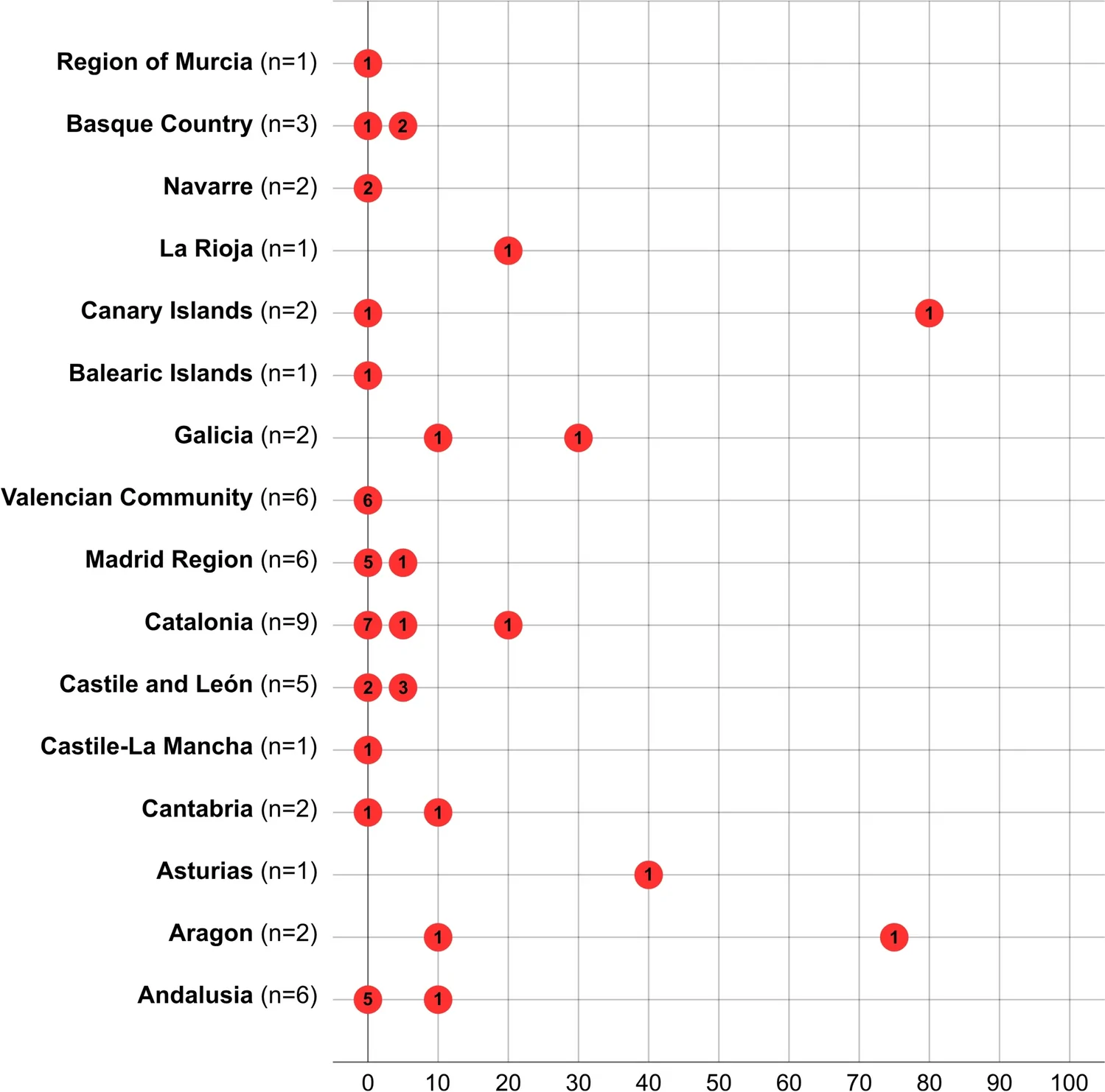
Estimated percentage of patients with cholangiocarcinoma and gallbladder cancer eligible for next-generation sequencing (NGS) who did not undergo the procedure due to center limitations. The number within each point indicates the number of respondents with that same answer. The total number of respondents per autonomous community is shown in parentheses

Despite these high indication rates, the proportion of eligible patients who did not undergo NGS testing due to local limitations varied considerably among respondents (Fig. 4). Most responses fell within the lower range (0–10%). Intermediate estimates (10–30%) were also reported by several respondents. However, higher values were observed in specific cases, such as 40% in Asturias, 75% in Aragon, and up to 80% in the Canary Islands.

With respect to the clinical utility of NGS, respondents reported that molecular findings influenced treatment decisions with varying frequency across respondents (Supplementary Fig. 2). Most responses fell within the low-to-intermediate range (10–40%). Some respondents reported higher impact estimates, exceeding 60% and reaching 100% in individual cases. These higher values appeared in several autonomous communities, including Castile and León, Catalonia, Andalusia, Community of Madrid, and the Canary Islands, but no consistent regional pattern emerged. Overall, responses varied significantly both across and within autonomous communities. For instance, in Catalonia, Community of Madrid, and Valencian Community, estimates ranged from low to high.

### Genetic counseling for NGS interpretation

Among the 48 respondents who reported performing NGS, 34 (71%) indicated that their centers offer access to the interpretation of complex molecular results, whereas 14 respondents (29%) reported that NGS is performed without access to specialized counseling.

Respondents from Catalonia, Cantabria, Castile-La Mancha, La Rioja, Navarre, and the Balearic Islands indicated that all the respondents who performed NGS in their communities had access to genetic counseling. Intermediate proportions were reported by respondents from the Community of Madrid (67%), Castile and León (60%), the Basque Country (67%), and the Valencian Community (50%).

Galicia was the only community in which none of the respondents reported access to genetic counseling. The results are shown in Table 1**.**

**Table 1:** Availability of genetic counseling to interpret complex NGS results in cholangiocarcinoma and gallbladder cancer

Autonomous community	Does your center offer genetic counseling to interpret complex NGS results in patients with cholangiocarcinoma?						
	No, because we do not do NGS		No, but we do NGS		Yes		Total (n)
	n	%	n	%	n	%	
Andalusia	1	17	2	33	3	50	6
Aragon	0	0	1	50	1	50	2
Asturias	0	0	0	0	1	100	1
Cantabria	0	0	0	0	2	100	2
Castile-La Mancha	0	0	0	0	2	100	2
Castile and León	0	0	2	40	3	60	5
Catalonia	0	0	0	0	9	100	9
Community of Madrid	0	0	2	33	4	67	6
Valencian community	0	0	3	50	3	50	6
Galicia	2	100	0	0	0	0	2
Balearic Islands	0	0	0	0	1	100	1
Canary Islands	0	0	2	100	0	0	2
La Rioja	0	0	0	0	1	100	1
Navarre	0	0	0	0	2	100	2
Basque Country	0	0	1	33	2	67	3
Region of Murcia	0	0	1	100	0	0	1
Total	3		14		34		51

%: percentage; n: sample size

### Participation in research and clinical trials in BTC

Among the 50 respondents, 27 (54%) reported participation in research projects or clinical trials related to CCA **(**Table 2**)**. Participation rates varied across respondents from different autonomous communities. All respondents from Aragon, Asturias, Cantabria, and Navarre reported involvement in research activities, while high participation levels were observed among respondents from Catalonia (78%), the Community of Madrid (67%), and the Valencian Community (67%). In contrast, respondents from Galicia, Castile-La Mancha, the Balearic Islands, La Rioja, and Murcia did not report participation in research or clinical trials during the evaluation period. Moderate participation levels were described by respondents from Andalusia (33%), Castile and León (20%), the Canary Islands (50%), and the Basque Country (33%).

**Table 2:** Participation of centers in research or clinical trials related to cholangiocarcinoma and gallbladder cancer

Autonomous community	Does your center participate in research or clinical trials to improve the diagnosis and treatment of cholangiocarcinoma?				
	No		Yes		Total (*n*)
	n	%	n	%	
Andalusia	4	67	2	33	6
Aragon	0	0	2	100	2
Asturias	0	0	1	100	1
Cantabria	0	0	2	100	2
Castile-La Mancha	1	100	0	0	1
Castile and León	4	80	1	20	5
Catalonia	2	22	7	78	9
Community of Madrid	2	33	4	67	6
Valencian community	2	33	4	67	6
Galicia	2	100	0	0	2
Balearic Islands	1	100	0	0	1
Canary Islands	1	50	1	50	2
La Rioja	1	100	0	0	1
Navarre	0	0	2	100	2
Basque country	2	67	1	33	3
Region of Murcia	1	100	0	0	1
Total	23		27		50

%: percentage; n: sample size

## Discussion

This study offers a comprehensive and up-to-date overview of how NGS is implemented in the management of digestive tumors, specifically in CCA and GBC, on the basis of the experience of specialists across Spain. It reveals a high level of NGS adoption, with most respondents reporting access to NGS for solid tumors, and 93% indicating its use in CCA.

These findings are consistent with international recommendations emphasizing comprehensive molecular profiling in advanced CCA. Current clinical guidelines, including those from the ESMO, recommend the use of NGS to identify actionable genomic alterations and guide therapeutic decision-making [13, 15]. Molecular profiling is particularly relevant given the increasing availability of targeted therapies directed against alterations such as FGFR2 rearrangements, IDH1 mutations and, recently, HER2 overexpression [13, 18].

In Europe, the implementation of molecular profiling has expanded substantially, although disparities remain due to differences in infrastructure, reimbursement, and institutional resources [19–21]. These disparities may influence the integration of precision oncology and contribute to variability in clinical practice.

The widespread assessment of FGFR2 and IDH1 alterations observed in this study aligns with international and national recommendations, which identify these alterations as key therapeutic targets in CCA [13, 18, 22]. Conversely, the lower use of expanded genomic panels suggests variability in the depth of molecular characterization, which may limit the identification of additional clinically relevant alterations [23].

Guidelines emphasize the importance of comprehensive molecular profiling, including both DNA- and RNA-based approaches, to ensure the detection of actionable genomic alterations [13, 22, 23]. Operational barriers such as tissue availability, sample quality, and a lack of standardized workflows remain as important challenges in routine clinical implementation [13, 19].

The timing of NGS testing varied across the participating centers, with most respondents reporting testing in advanced disease. Although this reflects current clinical practice, earlier molecular testing at diagnosis may facilitate timely therapeutic decision-making and improve patient access to targeted therapies and clinical trials [18, 22].

Despite high eligibility rates, the respondents reported that a proportion of patients did not undergo NGS testing because of local barriers. These findings are consistent with the European data highlighting infrastructure limitations, unequal access to molecular diagnostics, and organizational barriers as key factors influencing implementation [19–21].

However, Spain has structures in place that can help mitigate these inequalities. A prime example is the program offered by the Spanish Cooperative Group for the Treatment of Digestive Tumors (TTD), with more than 150 national hospitals and 8 international centers, through the RETUD registry (“Registro Español de TUmores Digestivos)”, which enables participating centers to perform centralized, cost-free NGS testing for BTC patients, including RNA sequencing in tissue and liquid ctDNA testing, regardless of local technical capabilities [24].

The clinical relevance of molecular profiling is underscored by the availability of approved targeted therapies in cholangiocarcinoma. FGFR inhibitors such as pemigatinib and futibatinib and the IDH1 inhibitor ivosidenib have demonstrated clinically meaningful benefits in molecularly selected patients. Notably, for example, reimbursement for ivosidenib in patients with IDH1-mutated refractory CCA was approved in March 2025, and this survey was performed in May 2025. This could have impacted the clinical utility of NGS reported by respondents. Emerging therapeutic strategies targeting HER2 and other molecular alterations may further expand treatment options in the near future [25]. For example, zanidatamab was approved by the EMA for HER2 (IHC3 +)-refractory BTC in June 2025 but still does not have approval for reimbursement in Spain.

The results of this study are consistent with those of other national studies. The established implementation of NGS in lung cancer supports its viability for less prevalent tumors such as CCA [26]. SEOM-SEAP consensus statements highlight the importance of identifying alterations in IDH1, FGFR2, and HER2 and advocate for the standardization of molecular diagnosis [18]. Furthermore, national reviews identify logistical and training barriers and propose algorithms tailored to the Spanish healthcare infrastructure [27]. Economic analyses in lung cancer also demonstrate the cost-effectiveness of NGS compared with Sanger sequencing methods [28], reinforcing the advisability of its expansion in CCA/GBC. These approvals exemplify the clinical implications of appropriate molecular profiling and support the incorporation of NGS into routine decision-making. Emerging therapeutic options, such as HER2-directed therapies [25] and BRAF-targeted strategies [29], are under active investigation and may further expand the therapeutic landscape in the near future. The increasing participation of Spanish researchers in international panels on the molecular biology of CCA and applications of NGS [13] confirms Spain’s emerging role in this field.

Additionally, real-world studies and economic analyses have demonstrated the clinical utility and cost-effectiveness of NGS-based molecular profiling, supporting its incorporation into routine oncologic care [14].

This study has several strengths, including its multicenter design, the involvement of specialists from various autonomous communities, and the provision of original data regarding a low-incidence/prevalence tumor. The territorial analysis enables the identification of potential specific inequities in access to molecular diagnosis, laying a solid groundwork for future initiatives aimed at standardizing and improving equity.

Importantly, some of the variability observed between centers may reflect differences in how molecular testing is implemented across specialties and institutions. In current BTC workflows, molecular characterization may encompass comprehensive NGS-based profiling (using DNA or RNA assays) [23] as well as biomarker-driven techniques such as immunohistochemistry, in situ hybridization, or PCR-based methods [30]. Since the analytical performance and clinical suitability of each platform may depend on the specific alteration being investigated (e.g., certain rearrangements, amplifications, or protein expression readouts), centers may adopt heterogeneous testing algorithms that combine broad profiling with targeted confirmatory assays. Therefore, clearly distinguishing NGS (DNA or RNA) from other molecular diagnostic modalities is essential when real-world implementation data are interpreted and harmonized diagnostic pathways are designed.

We did not address whether molecular testing was performed locally or via a clinical trial prescreening platform. Additionally, we did not review whether molecular testing was performed in clinical practice and locally in the same hospital or by referral to a centralized regional platform with or without certification. However, this study has several limitations. The questionnaire used was not formally validated, the responses were based on expert perceptions, and the open selection of participants could lead to bias. Additionally, the exploratory nature of the study, which did not involve a formal sample size calculation, limits its generalizability. Nevertheless, the consistency observed across different communities and the agreement with findings from other studies support the overall validity of the analysis.

## Conclusions

This study revealed that NGS is now well established for the diagnosis of CCA in Spain, with FGFR2 and IDH1/2 being the most frequently analyzed biomarkers in laboratories. However, variability in the implementation of molecular testing persists, particularly in terms of the timing of testing, integration into clinical workflows, and access to specialized resources such as genetic counseling. National facilitation of centralized molecular testing may help reduce disparities and support broader implementation of precision oncology. These findings highlight the need for standardized molecular testing strategies to ensure equitable access to targeted therapies and optimize clinical outcomes.

## Supplementary Information

Below is the link to the electronic supplementary material.

Supplementary file1 (JPG 1001 KB)

Supplementary file2 (DOCX 710 KB)

## Data Availability

Not applicable. All additional information can be found in the Supplementary material.
