# Supplementary material for: Current patterns of next-generation sequencing testing in biliary tract cancer patients in Spain
Source: Clin Transl Oncol. 2026 May 28;28(11):5344–54. doi: 10.1007/s12094-026-04397-3 (PMC13694501; doi:10.1007/s12094-026-04397-3)
Supplement: Supplementary file 1 [file 12094_2026_4397_MOESM2_ESM.docx]

**Supplementary material**

**Supplementary Figure 1.** Geographic distribution of access to next-generation sequencing (NGS) techniques for cholangiocarcinoma and gallbladder surgery in Spain. The calculations were performed considering the number of centers for which access to NGS techniques was indicated (n=54). It does not represent access to an NGS per region, as in autonomous communities, the NGS performance is centralized in 1–2 centers for all health institutions.

**Supplementary Figure 2:** Estimated percentage of patients in whom next-generation sequencing (NGS) results altered treatment decisions.

The number within each point indicates the number of respondents with that same answer. The total number of respondents per autonomous community is shown in parentheses.
